# Abnormal Parietal Function in Conversion Paresis

**DOI:** 10.1371/journal.pone.0025918

**Published:** 2011-10-24

**Authors:** Marije van Beilen, Bauke M. de Jong, Esther W. Gieteling, Remco Renken, Klaus L. Leenders

**Affiliations:** 1 Department of Neurology, University Medical Center Groningen, University of Groningen, Groningen, The Netherlands; 2 BCN NeuroImaging Center, University Medical Center Groningen, University of Groningen, Groningen, The Netherlands; Cuban Neuroscience Center, Cuba

## Abstract

The etiology of medically unexplained symptoms such as conversion disorder is poorly understood. This is partly because the interpretation of neuroimaging results in conversion paresis has been complicated by the use of different control groups, tasks and statistical comparisons. The present study includes these different aspects in a single data set. In our study we included both normal controls and feigners to control for conversion paresis. We studied both movement execution and imagery, and we contrasted both within-group and between-group activation. Moreover, to reveal hemisphere-specific effects that have not been reported before, we performed these analyses using both flipped and unflipped data. This approach resulted in the identification of abnormal parietal activation which was specific for conversion paresis patients. Patients also showed reduced activity in the prefrontal cortex, supramarginal gyrus and precuneus, including hemisphere-specific activation that is lateralized in the same hemisphere, regardless of right- or left-sided paresis. We propose that these regions are candidates for an interface between psychological mechanisms and disturbed higher-order motor control. Our study presents an integrative neurophysiological view of the mechanisms that contribute to the etiology of this puzzling psychological disorder, which can be further investigated with other types of conversion symptoms.

## Introduction

The etiology of medically unexplained symptoms such as conversion disorder is poorly understood. Neuroimaging studies of conversion paresis have not yet resulted in a commonly accepted theory about the functional neuroanatomy of this disorder. Because researchers have used different control groups, different behavioral tasks and different statistical comparisons, the results have been difficult to generalize between studies. However, by combining the previously reported control groups, tasks and statistical comparisons in various studies, we extracted common neurophysiological abnormalities specific for this disorder. This enabled us to formulate a hypothetical, neurophysiological model that applies to conversion disorder in general.

Conversion disorder encompasses medically unexplained, physical symptoms that are believed to develop unintentionally in reaction to psychological and environmental factors such as trauma or daily stressors [1]. This disorder inspired Freud to develop his theories of consciousness, autobiographic memory and self-reflection [2], which became the cornerstone of modern psychology. Current criteria for conversion disorder include one or more symptoms affecting voluntary motor or sensory function that cannot be attributed to a neurological disorder. Symptoms suggest a medical condition with a behavioral presentation mimicking various types of neurological symptoms including tremor, dystonia, pseudo-epileptic insults, sensory loss and paresis. Unlike a factitious disorder or malingering, conversion symptoms are not deliberately produced. According to cognitive theories on conversion disorder [3], symptoms are unintentionally “produced” by activating inappropriate cognitive schemata or memories while simultaneously inhibiting appropriate ones. The outcome, but not the process itself, is consciously perceived by the patient as a physical symptom.

The current study concerns a subtype of conversion disorder: conversion paresis. From a neurological perspective, abnormal cerebral activity in circuitry implicated in higher order motor control [4] would be expected in this disorder. Furthermore, psychological factors may influence movement preparation in relevant motor areas such as the prefrontal cortex (implicated in aspects of willed action) and the parietal cortex (implicated in the integration of psychological and environmental information). The preparation of internally driven movement is normally located in secondary cortical motor areas [5], [6] as well as in the cingulate cortex [7], [8].

Neuroimaging studies [9]–[15] (see Table 1 for an overview) investigating the neural activity in Conversion Paresis have shown anterior cingulate hyperactivation compared to normal controls during movement execution of the paralyzed limb. This was originally interpreted as inhibiting normal movement [12]. Subsequent theories focused on the self-monitoring function of the anterior cingulate gyrus [10], [15], [16]. However, the increases in cingulate activation reported in previous research are not consistently present in all experimental paradigms (this applies to both conversion paresis and feigners [13], [14], [17], [18]). A recent review [19] and a neuroanatomical study [20] discussed the original interpretation of increased anterior cingulate activation. Similarly, prefrontal activation has been reported as being increased or decreased in patients, depending on the paradigm used. Prefrontal activation was consistently decreased in paradigms based on motor execution [12]–[14]. This is consistent with the idea that conversion paresis is characterized by impaired control of conscious motor will (willed action), accompanied by abnormal levels of prefrontal motor function [13]. During motor imagery [11], increased activation of the prefrontal cluster was reported, while in a later study the same group found more specifically that the dorsolateral part of the prefrontal cortex was connected with parts of the motor system that are involved in action planning [21].

**Table 1:** Summary of fMRI results of abnormal cortical activation in conversion paresis.

Author^1^	CP	NC	FC	Task	Activation^2^	Method^3^
Marshall (1997)	n = 1 L arm/leg (leg fixated)			movement execution and –preparation	+ C ACC (BA 24/32)	A-UA
					+ C frontal pole (BA 10)	
					− I prim sensory cortex	AM-AP
					− I prim motor cortex	
					− I supramarginal (BA 40)	
					− C inf temp cortex (BA 20/28)	
Spence (2000)^4^	n = 2 L arm	n = 6 HC	n = 2 L arm	movement execution affected side	− I prefrontal areas (BA 9/46)	CP-NC
					− I prefrontal areas (BA 9/46)	CP-FC
Vuilleumier (2001)	n = 7			passive vibration	+ C prefrontal (BA 6,8,9,46)	A - R
	4*L arm (/+leg)				− C med occipital (BA 19/18)	A – UA(R)
	3*R arm (/+leg)				+ I prefrontal (BA 9/46,46,9,8)	
					− C,I inf occipital (BA 18)	
					+ C,I prim somatosen. (BA1/2,5)	
					− I lingual gyrus (BA 18)	
					C somatosensory ass (BA7)	
Burgmer (2006)^5^	n = 4	n = 7		movement execution and –observation	no abnormal activation^6^	A-R
	3*L arm + leg				absent activity motor cortices	
	1*R arm + leg					
						
de Lange (2006)	n = 8			implicit imagery	+ medial frontal^6^	A-UA
	4*R arm				+ parietal operculum	
	4* L arm				+ superior temporal sulcus	
					+ superior temporal gyrus	
de Lange (2008)	n = 7			explicit imagery	no abnormal activation^6^	A-AU
	4*R arm			implicit imagery	+ gyrus rectus	
	3*L arm				+ med frontal gyrus	
					+ sup frontal gyrus	
					+ sup temp gyrus	
Stone (2007)	n = 4 R leg		n = 4	movement execution	+ C Paracentral lobe (BA 4)	A-UA
					+ C,I putamen	
					+ C insula	
					+ C,I inf. frontal gyrus	
					+ C,I lingual (BA 18)	
					+ C sup parietal gyrus (BA7)	
					− I paracentral lobe (BA 4)	
					− I prefrontal areas (BA 10,11 46)	
					+ C paracentral gyrus (BA 4)	A-UA
					+ C SMA (BA 6)	
					+ I cerebellum	
					− I paracentral lobe (BA 4)	
					− I parahippocampal (BA 29)	
					− C cerebellum	
					− I mid occipital (BA 19/39)	
					− C sup parietal lobe (BA7)	

1: first author; 2: + = increased activation, − = decreased activation, I = ipsilateral; C = contralateral, 3: A = Affected side, UA = Unaffected side, AM = Affected side movement, AP = Affected side movement preparation, UA(R) = Affected side after recovery, R = Rest condition,; 4: PET; 5: spastic paresis, 6: authors did not provide BA.

Although the studies discussed above appear to have inconsistent results, this is probably because conversion paresis was investigated with different paradigms: different tasks (e.g. motor execution, imagining, or observation) and different control groups (e.g. healthy subjects moving normally or healthy subjects feigning conversion paresis [13], [17], [22]. In a complex psychological condition such as conversion paresis, differing neuroimaging results could also be expected. Nevertheless, initial neuroimaging results have shown that cerebral activation in conversion paresis is abnormal, although a coherent underlying mechanism of neuronal dysfunction needs to be elucidated.

### Methodological aspects of previous studies

The incomparability of previous studies appears to be related to the neuroimaging methods and paradigms used. There are two methodological aspects that affect the comparability of such studies.

First, functional neuroimaging as a method in general does not discriminate between abnormal task-evoked cerebral activity which causes a symptom, and abnormal activity which is a result of a symptom (such as abnormal movement in conversion paresis. When they are moving unnaturally, healthy subjects all show seemingly abnormal cerebral activity. When abnormal movements are performed, sensory feedback changes. To overcome the effects of sensory feedback, paradigms employing movement imagery have been applied. But even then, when studying conversion paresis patients compared to healthy controls, researchers inevitably find (a) abnormal activity associated with a cerebral state, especially in a person suffering from conversion paresis, and (b) abnormal cerebral activation that is the result of not moving (or imagining movement) normally, regardless of the cause (i.e. intentional or unintentional paresis).

Second, intentional and unintentional abnormal movement results in identical abnormal cerebral activation patterns (with respect to activation that is related to the act of moving, for example sensory feedback). Two underlying issues are involved.

1. Without comparison with healthy controls who intentionally feign paresis, it is impossible to distinguish between cerebral correlates of abnormal movement output caused by conversion paresis and the cerebral correlates of identical abnormal movement output caused by healthy, intentional, mechanisms. Therefore, the neurophysiological abnormalities that are specific for conversion paresis would not be identified.
2. Abnormal activation that causes conversion paresis includes hemisphere-specific activation, which is independent of symptom lateralization, in addition to activation contralateral to the affected side. In previous studies, a common strategy was to assess within-group differences by contrasting the affected and unaffected limb. The resulting hemispheral difference was subsequently compared with the hemispheral difference in a healthy control subjects. This strategy, however, may neglect the effects of hemisphere-specific activation, which is present in conversion paresis regardless of symptom lateralization. As previous studies addressed both left- and right sided symptoms, half of the data were flipped in order to obtain a homogeneous group for analysis with all abnormal hemispheral function contralateral to the affected limb [10], [14]. Although this is a good methodological strategy, it also conceals hemisphere-specific activity. Essentially, flipped data and within-group contrasts specifically reveal abnormalities in contralateral activation, while unflipped data and between-group contrasts may reveal additional hemisphere-specific abnormalities in cerebral activation.

In sum, it remains unknown what neurophysiological abnormalities in conversions paresis are specific for unintentional paresis. Moreover, hemisphere specific activity has not been revealed by previous paradigms.

### Present study

In our study we used 4 different types of data-analyses to focus on the unknowns described above. Our first, general, research question was the following: do the comparisons of patients with feigners and controls, reveal meaningful neurophysiological abnormalities when they are not controlled for within-patient factors? More specifically, we addressed two additional hypotheses. First, based on the relevant literature, we expected to find increased anterior cingulate activation and decreased prefrontal activation in within-group contrasts. Second, reasoning from a neurological perspective, we expected to find conversion paresis-specific cerebral activity, which would indicate a) impaired willed action (i.e. decreased prefrontal activation) to account for the idea that symptoms occur unintentionally, b) abnormal parietal activity in between-group contrasts, associated with the cognitive theories of impaired activation of movement schemata in conjunction with environmental cues, and c) increased activity reflecting abnormal movement initiation in motor areas responsible for movement abnormal preparation (i.e. Premotor Cortex (PM) activity and Pre- Supplementary Motor Area (SMA)) in both patients and feigners.

In our study, we addressed these questions as follows. First, we analyzed both standard within-group and additional between-group contrasts separately in the same data. Second, we analyzed both flipped and unflipped data. Third, we compared the cerebral correlates of conversion paresis (unintentional) abnormal movement to both feigned (intentional) abnormal movement and normal movement. Fourth, we also investigated both movement imagery and execution. This not only resulted in an interesting overview of abnormal cerebral activation in conversion paresis, but also yielded a large amount of data with complex interrelationships.

Although whole-brain analyses were performed, for purposes of clarity and brevity we limited the description and discussion of the results in the text mainly to the areas described above: the cingulate cortex, dorsolateral prefrontal cortex (DLPFC), motor areas (motor cortex, premotor cortex, supplementary motor area (SMA)), and supramarginal gyrus. The complete results (significant at cluster level, see below) are presented in the tables.

## Materials and Methods

### Ethical statement

This study was approved by the ethical committee of the University Medical Centre Groningen. All participants gave written informed consent and were treated according to the declaration of Helsinki.

### Subjects

See Table 2 for subject characteristics. Originally 5 groups were included: 21 normal controls, right-sided feigning paresis (n = 7) and left-sided feigning paresis (n = 6), right-sided conversion paresis (n = 6) and left-sided conversion paresis (n = 4) (see statistical analyses for the method used to merge the left- and right sided groups in analyses A and B). These groups were merged into 3 groups after flipping the data for analyses C and D (see below). In the Methods and Results sections, these groups are referred to as ‘patients’, feigners’, and ‘normal controls’ for reasons of brevity. All patients suffered from flaccid paresis; dystonic paresis was excluded. One patient was left handed. The clinical diagnosis was made after neurological examinations that were performed according the following protocols:

1. Clinical exam. Special attention was given to inconsistencies in muscle strength during the clinical exam and between the actual exam and informal behavior such as getting dressed, opening the door and shaking hands. In patients with paresis of the leg, Hoovers sign was included. Symptoms were consistently and congruently present in three patients; others experienced periods without symptoms, but showed symptoms during scanning procedures.
2. Neurophysiological exam. All patients were examined with Motor Evoked Potential (MEP) with normal results.
3. Biological tests. Extensive blood tests were performed, including hematology, electrolytes, kidney and liver functioning and glucose. All patients had normal results.
4. Psychological exam. Psychogenic factors were confirmed by an expert neuropsychologist (M.B.) focusing on the existence of a relationship between a psychological life event, daily hassles and neurological symptoms. Psychological tests regarding dissociative symptoms, psychological life events, psychological complaints (including anxiety and depression, personality pathology, and coping styles) and attentional disorders were administered. The latter results were not included in this paper; see [23]–[25] for an overview of the tests and results.
5. Clinical imaging (cerebrum and myelum) was performed on all patients with 1.5T MRI using standard T1/T2-FLAIR sequences. In addition, the anatomical T1 images obtained with 3T MRI in this functional imaging study were inspected for all subjects by an expert neurologist (B.M.J.) to check for organic damage. One additional patient was excluded due to structural brain damage. All other patients (n = 9 remained) had normal results.

**Table 2:** Demographic and illness variables.

		NC (n = 21)	CP (n = 9)	FC (n = 13)
Age		43.3 (SD 9.7)	44.8 (SD 11.4)	45.1 (SD 12.7)
Sex	Male	4 (19%)	2 (20.2%)	9 (20.6%)
	Female	17 (81%)	7 (77.8%)	4 (71.4%)
Paralysis				
	Side		R:5, L:4	R:7, L:6
	Severity		25 (18.7)	27.1 (17.6)

Patient	Age	duration*	Fluctuating	Severity %**	Comorbidity***	Rating scan movement
1	50	5	yes	20	sexual abuse	3
2	56	1	yes	0	loss of spouse	1
3	51	8	no	20	marital distress	4
4	18	2	yes	40	N.I.****	4
5	43	2	yes	10	car accident	1
6	52	1	yes	20	sexual abuse	3
7	51	0.3	no	20	work disstress	2
8	42	0.5	no	65	somatization disorder.	4
9	40	3.0	yes	30	illness child	3
Mean (sd)	45 (11)	2.5 (2.5)		25 (19)		

*years;

**Percentage of normal functioning according to patient;

***Psychiatric or Psychological co-morbidity;

****Not identified.

### Procedures

Handedness was assessed according to the Dutch Handedness Questionnaire [26]. The ability to perform mental imagery was assessed by the Vividness of Movement Imagery Questionnaire (VMIQ) [27], where low scores indicate better performance. Further assessments included a neurological examination by a movement disorder neurologist (K.L.L.). The T1 weighted MRI scans were analyzed by an experienced neurologist (B.M.J.) to assess possible cerebral pathology. Informed written consent was obtained according to the declaration of Helsinki and the study was approved by the Medical Ethical Committee of the UMCG.

All groups performed four tasks: execution and imagination of flexion/extension movements of the right and left wrist separately [28]. Prior to the experiment, subjects practiced the tasks for 2 minutes outside the MRI scanner. They were instructed to move their wrist, but according to a 0.5 Hz pace which was indicated by a visual cue (a flickering dot on the screen). The movements were performed in a vertical plane with stretched fingers. The instructions for execution were “try to move your hand as closely as possible according to the rhythm of the dot (movement shown by the instructor and practiced by the patient)”. The instructions for imagery were “imagine that you are moving your hand in an identical manner and according to the same rhythm compared to the movement execution condition”.

During scanning, patients were presented with visual, written, instructions that indicated the condition: ‘Right, Move’; ‘Left, Move’; Right, Think’; ‘Left, Think’; ‘Rest’. Prior to the scanning procedure, feigners received the following general instruction: “in this experiment, while you are in the MR scanner you have to simulate a paresis of your right/left hand as you would do if you had to convince a medical examiner that your hand is partly paralyzed, feels heavy and is difficult to move”.

During scanning the forearms were positioned in pronation on pillows, and we made sure subjects did not touch the scanner. Their limbs were not within their field of view. Four task conditions were defined: movement execution in the affected hand (Execution affected), movement execution in the unaffected hand (Execution unaffected), movement imagery in the affected hand (Imagery affected), and movement imagery in the unaffected hand (Imagery unaffected).

All tasks were preceded by a rest condition. All conditions had a duration of 30 seconds. Three runs were performed lasting 12 minutes each. During each run, 12 response blocks (one task per block) were scheduled. For each subject, tasks were presented in a random but balanced order (i.e. all four conditions were presented three times each). All subjects were video-taped during scanning. Severity of paresis in patients and feigners during task execution was rated using a rating scale of 1–5 on the degree of flexion and extension of the wrist and the degree of stretching of the fingers by two independent expert neurologist raters (1 = none, 2 = minor movement, 3 = partial extension/flexion of the hand, 4 = slowed or stiff full extension/flexion of the hand, 5 = normal full extension/flexion of the hand).

### Functional imaging

Cerebral activation in subjects was scanned using a 3 Tesla Philips MRI scanner (Best, the Netherlands). The following pulse sequence parameters were used: single shot EPI; 46 slices; 3.5 mm slice thickness; no gap; 224×224 mm field of view; 64×64 scan matrix; transverse slice orientation; repetition time 3000 ms; echo time 35 ms; flip angle 90°. Three runs of 240 brain volumes each were acquired, i.e. 10 volumes per 30 seconds condition block. In addition, a T1-weighted whole brain anatomical image was acquired (resolution 1×1×1 mm).

### Statistical analysis

Patients and feigners were compared for the degree of movement during the task (while scanning) with a two-sample t-test (two-tailed). Patients and all normal controls were compared using the VMIQ questionnaire with a two-sample t-test (two-tailed).

We performed four types of data-analyses on the same group of subjects to reveal different aspects of abnormal activation. Note that in analyses B, C and D, both flipped and unflipped between-group contrasts were not controlled for within-group comparisons for reasons explained in the introduction. We made direct comparisons between groups for each condition, e.g. affected side patients versus affected side normal controls for the condition ‘Execution affected’.

For analyses A and B, the data were not flipped. When flipping the data, it is assumed that right- and left-sided paresis will show identical effects (contain identical variances). We allowed a possible different effect size for left and right affected groups (at the cost of two degrees of freedom) by initially defining five groups instead of three (this minimizes error variance.) Therefore, two-way between-group ANOVA was used. Factor 1 (group) contained five levels (normal controls, right feigners, left feigners, right patients, and left patients), and factor 2 (condition) contained four levels (Execution affected, Execution unaffected, Imagery affected, Imagery unaffected). To limit the number of comparisons, the left- and right sided groups were merged in the definition of the contrasts. Thus, note that also in unflipped data (analyses A and B), only three (not five) comparisons were made, with group sizes n = 22 for normal controls, n = 13 for feigners, and n = 9 for patients.

#### A. Within-group contrasts

Affected and unaffected sides were compared for each subject. The following three contrasts were investigated within each group: A1) right hand versus left hand for the normal control group, A2) affected versus unaffected hand in the patient group, and A3) affected versus unaffected hand in the feigner groups.

#### B. Between-group contrasts in unflipped data

In this contrast, unflipped data were analyzed to convey the hemisphere-specific activations. For this purpose, the groups of left- and right-sided paresis were included as one group (n = 9), and the groups of left- and right-sided feigned paresis were included as one group regardless of symptom side (n = 13). In these three groups, two-way between-group ANOVA was used. Factor 1 (group) contained 5 levels (normal controls, right feigners, left feigners, right patients, and left patients), and factor 2 (condition) contained 4 levels (Execution affected, Execution unaffected, Imagery affected, Imagery unaffected). The following contrasts were investigated in all four task conditions: B1) normal controls versus patients; B2) normal controls versus feigners, and B3) patients versus feigners.

For analyses C and D, the data were flipped. For this purpose all parametric maps from first level analysis of the left-sided pareses (4 patients, 6 feigners) were flipped prior to second level analysis. As a result all patients and feigners ‘became affected’ on the right side. Two groups of patients with conversion paresis (with either right- and left-sided paresis) were merged into one group of n = 9 patients by defining affected ((n = 5 right-sided)+(n = 4 left-sided) = n = 9 affected side)) and unaffected ((n = 5 left-sided)+(n = 4 right-sided) = n = 9 unaffected side)) sides. Similarly, two groups feigning paresis (including right- and left-sided paresis) were merged into one group of n = 13 feigners by defining affected ((n = 7 right-sided)+(n = 6 left-sided) = n = 13 affected side)) and unaffected ((n = 7 left-sided)+(n = 6 right-sided) = n = 13 unaffected side)) sides. Note that only three (not five) comparisons were made in analyses C and D, with group sizes n = 21 for normal controls, n = 13 for feigners and n = 9 for patients.

#### C. Between-group contrasts in flipped data

Two-way between-group ANOVA was used. Factor 1 (group) contained 3 levels (normal controls, patients, feigners) and factor 2 (condition) contained 4 levels (Execution affected, Execution unaffected, Imagery affected, Imagery unaffected). The following contrasts were investigated: C1) normal controls versus patients; C2) normal controls versus feigners, and C3) patients versus feigners.

#### D. Conjunction analysis

To identify overlap in the resulting activation increases in contrasts formed in C, we performed a conjunction analysis whereby we investigated which anatomical areas were differently activated in normal controls compared to both patients and feigners. This isolates abnormal activity related to abnormal movement output of patients and feigners in general, regardless of its cause (see introduction). The contrast investigated here was D1) normal controls versus (patients and feigners). We also investigated what anatomical areas were abnormally activated in patients compared to both control groups to isolate abnormal activity specific for patients. This isolates abnormal activity specific for patients. The contrast investigated here was D2) patients versus (normal controls and feigners).

Spatial pre-processing and statistical analysis were carried out using Statistical Parametric Mapping (version SPM2) [29]. The functional images were realigned, co-registered to their own anatomical image, normalized to a standard brain (MNI) and subsequently smoothed with an isotropic Gaussian filter using an 8 mm Full Width-Half Maximum Gaussian kernel. Data were analyzed using a random effects model. At the first level (per subject), conditions were modeled as a box car convolved with the canonical hemodynamic response function. The contrasts of the movement and imagining conditions for each hand in each condition (versus baseline) were fed into the second level.

**Figure 1:**
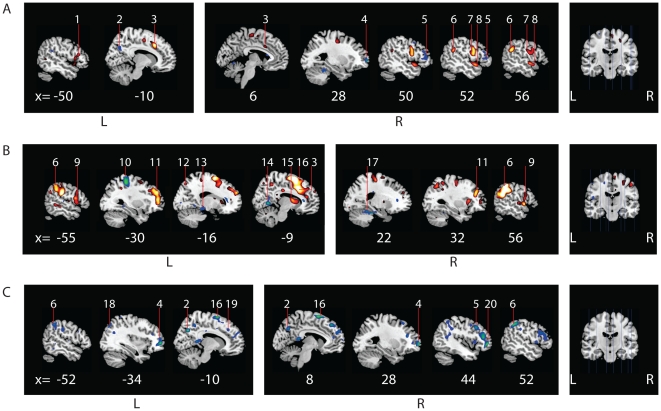
Cerebral activation patterns during movement execution in the affected hand for patients versus normal controls and patients versus feigners. Flipped data for movement execution in the affected side is shown for (A) patients versus normal controls, (B)feigners versus normal controls and (C) feigners versus patients. Figures were created with MRIcron, template Ch2better.nii.gz., without restriction of number of voxels. X-coordinates (in mm) for each saggital section are listed below. Numbers indicate clusters that were significant at cluster-level (P<0.05, whole-brain corrected at cluster level; k>200) and are also listed in the Tables. Note that this Figure also displays activity (not numbered) significant at an uncorrected threshold, to preserve explorative qualities of the study. A: Increased activation (red, t = 3.16 to yellow, t = 7) and decreased activation (blue t = 3.16 to green, t = 6.0) in patients versus normal controls. B: Increased activation (red, t = 3.16 to yellow, t = 8) and decreased activation (blue t = 3.16 to green, t = 6.0) in feigners versus normal controls. C: Decreased activation in patients vs feigners (blue t = 3.16 to green, t = 7.0). 1 = inferior frontal gyrus, 2 = precuneus, 3 = medial cingulate cortex, 4 = frontal pole, 5 = ventral lateral prefrontal cortex, 6 = supramarginal gyrus, 7 = premotor cortex, 8 = superior temporal cortex, 9 = superior frontal operculum, 10 = primary somatosensory cortex, 11 = dorso lateral prefrontal cortex, 12 = somatosensory association cortex, 13 = parahippocampal gyrus, 14 = primary/secondairy visual cortex, 15 = nucleus caudatus, 16 = SMA (in B extending into cingulate cortex), 17 = cerebellum, 18 = superior parietal cortex, 19 = frontal eye fields, 20 = orbitofrontal cortex. L = left, R = right.

Clusters of activation that reached statistical significance at cluster-level P<0.05 (corrected for multiple comparisons, initial threshold p<0.001 (T>3.14) are discussed. In our data set, cluster size (k)>200) for significant clusters. Note that Figure 1 also contains activity significant at an uncorrected threshold of p<0.001 T>3.14 (see Figure 1).

## Results

### Behavioral data

A trend towards better performance in control subjects was found in the questionnaire on the capacity of movement imagery (VMIQ) between patients (141±78) and controls (including feigners; 92±50); (two-sample T-test, 2-tailed p = 0,057). Because this result was not significant, we did not include it in further data analyses. Most patients were able to move their affected hand to some degree, ranging from 10 to 90% of normal use, and feigners showed similar movement patterns during movement execution conditions. All patients tried to move the hand in question appropriately throughout the entire scanning sessions. No differences in the degree of weakness (feigned or otherwise) was found between feigners and patients according to the scanning performance ratings (Table 2: patients: mean = 2.6, sd = 1.1; feigners: mean = 2.6, sd = 1.0, two-sample T-test, 2-tailed p = 0.8).

### fMRI data

Distinct activation patterns for patients, feigners and normal controls were identified. Significant results were found in both movement execution and movement imagery conditions. Results from analyses A, B, C and D were presented in the Tables and Figures if they reached significance at cluster-level P<0.05 (FWE), corrected for the entire brain volume, cluster size (k)>200; voxel size 2×2×2 after normalization). Of these, the anatomical areas that were relevant according to the hypotheses described in the Introduction are also discussed in the text.

### A. Within-group contrasts: affected versus unaffected side

Normal controls showed increased activity related to hand movement in the primary and secondary motor areas contralateral to the motor activity (Table 3). This was consistent with the side where the movement took place. Feigners showed a similar pattern, but a decrease in SMA activity was found on the affected side compared to the unaffected side. Patients showed decreased activity in multiple prefrontal areas and increased activity in the contralateral primary motor cortex consistent with the expected movement-related activity. The increased anterior cingulate activity reported in the literature (see Introduction) was not present at our threshold, but appeared at a much lower threshold – uncorrected p = 0.05 (MNI x = 0, y = 38, z = 0, t = 1.65.

**Table 3:** Cerebral activation in within-group comparison Affected versus Unaffected* in patients and feigners) side during movement execution.

				Left			Right			
Contrast**	BA	k	x	y	z	t/Z	x	y	z	t/Z
**CP A>CP UA**										
1. primary motor cortex	4	455	−10	−8	52	6.4/5.9				
2. somatosensory ass. cortex	5	2024	−30	−30	54	7.8/7.0				
3. (sec) visual (ass) cortex	18/19	255					28	−68	2	4.6/4.4
**CP A<CP UA**										
1. prim. motor cortex	4	216					4	−20	52	4.4/4.2
2. somatosen. ass.cortex	5	1470					38	−28	58	7.8/7.9
3. DLPFC	9	785					48	40	8	5.1/4.8
4. DLPFC	45	903					38	22	8	5.0/4.7
5. medial frontal pole	10	203					8	60	34	4.5/4.3
6. insular cortex	43	220	38	−6	−4	5.3/5.0				
7. cerebellum		809	−2	−52	−44	5.6/5.3				
**FC A>CP UA**										
1. primary motor cortex	4	1366	−34	−22	52	5.4/5.1				
2. cerebellum		433					12	−56	−20	5.4/5.1
**FC A<CP UA**										
1. motor and somatosens. areas.	4,5,6	3333					36	−26	60	8/7.1
2. insula	43	360					38	4	14	5.4/5.1
3. cerebellum		1191	−22	52	−26	7.0/6.4				
**NC R>L**										
1. motor and somatosens. areas	4,5,6	4618	−38	−34	60	14.0/>8				
2. insula	43	622	−36	−20	16	5.5/5.2				
3. cerebellum							14	−54	−18	11.4/>8
**NC R<L**										
1. motor and somatosens. areas	4,5,6	4771					38	−28	58	11.3/>8
2. insula	43	768					18	−20	2	6.6/6.1
3. cerebellum		2029	−12	−54	−22	11.1/>8				

Coordinates refer to the voxels of maximum activation within significant clusters (P<0.05, whole-brain corrected at cluster level). Positive x, y, z coordinates (in mm) indicate locations right, anterior and superior to the middle of the anterior commissure. CP = conversion paresis, FC = feigning controls, NC = normal controls, A = Affected side, UA = Unaffected side, R = right side, L = Left side, BA = Brodmann area.

*Note that the data are unflipped. ‘Affected side’ can include both right and left hands in patients and feigners.

**Movement imagery showed no significant results at p = 0.001 for any of the groups.

### B. Between-group contrasts in unflipped data: hemisphere-specific activation

#### Movement Execution in the affected hand

Patients showed increased left cingulate cortex activation in comparison to normal controls, spreading bilaterally at a lower threshold (Table 4). The right supramarginal gyrus and DLPFC showed decreased activation in patients versus feigners, but not compared to normal controls. In feigners, bilateral supramarginal gyrus and left DLPFC were overactivated compared to normal controls.

**Table 4:** Cerebral activation in between-group comparisons in unflipped data: hemisphere-specific activation.

			Left				Right			
Contrast*	BA	k	x	y	z	t/Z	x	y	z	t/Z
AEX**										
CP>NC										
1. anterior cingulate gyrus	32	446	−8	20	42	4.3/4.2				
FC>NC										
1. supramarginal gyrus	40	309	−56	−42	38	4.2/4.2	54	42	46	4.0/3.8
FC>CP										
1. DLPFC	46	278					44	40	22	4.5/4.3
2. supramarginal gyrus	40	257					54	−40	46	4.3/4.2
AIM										
FC>NC										
1. primary somatosensory cortex	2	491					62	−18	28	4.4/4.3

Coordinates refer to the voxels of maximum activation within significant clusters (P<0.05, whole-brain corrected at cluster level). Positive x, y, z coordinates (in mm) indicate locations right, anterior and superior to the middle of the anterior commissure.

*CP = conversion paresis, FC = feigning controls, NC = normal controls, A = Affected side, UA = Unaffected side, BA = Brodmann area,

*CP = conversion paresis, NC = normal controls, FC = feigning controls;

**AEX = movement execution of the affected hand, AIM = movement imagery of the affected hand.

### C. Between-group contrasts in flipped data

**Figure 2:**
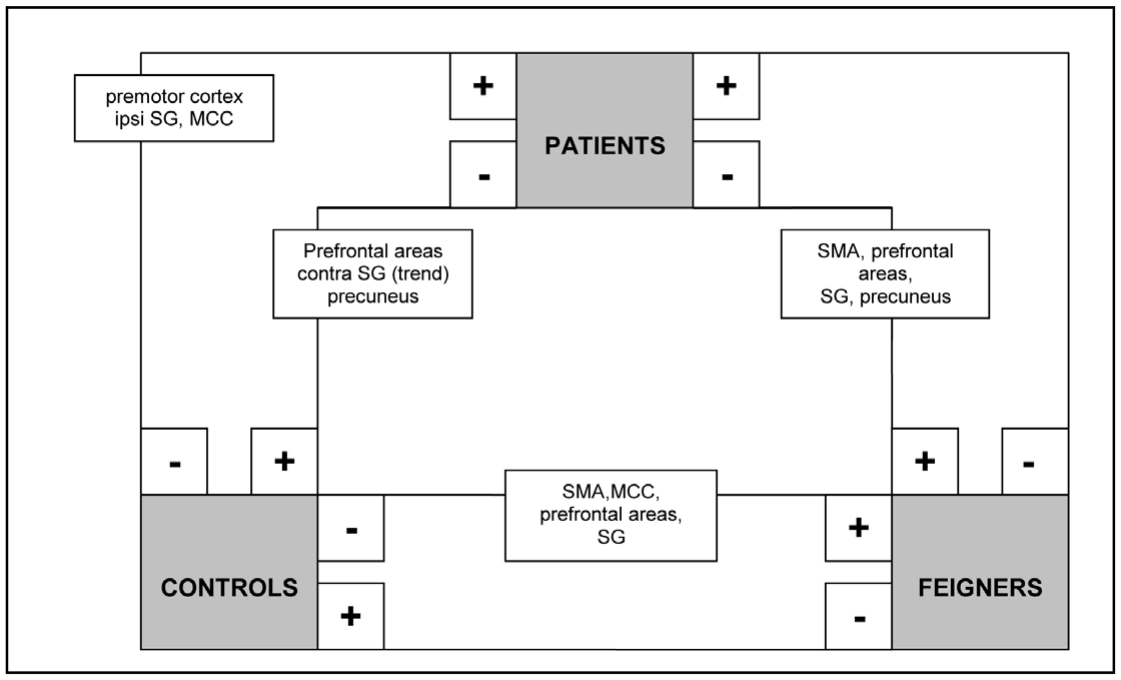
Schematic summary of significant clusters (P<0.05, whole-brain corrected at cluster level; k>200) in between-group comparisons (flipped data): movement execution in the affected hand (analysis C). + = significantly more activated than -.

**Figure 3:**
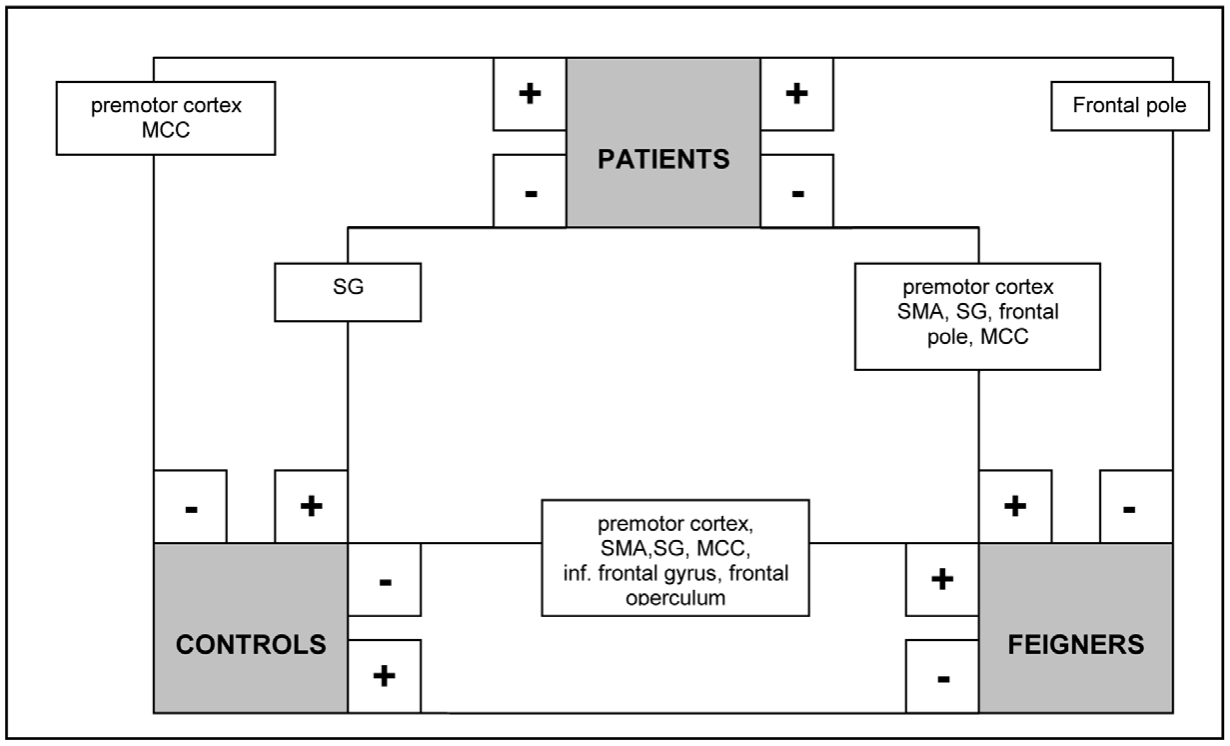
Schematic summary of significant clusters (P<0.05, whole-brain corrected at cluster level; k>200) in between-group comparisons (flipped data): movement execution in the unaffected hand (analysis C). + = significantly more activated than -.

**Figure 4:**
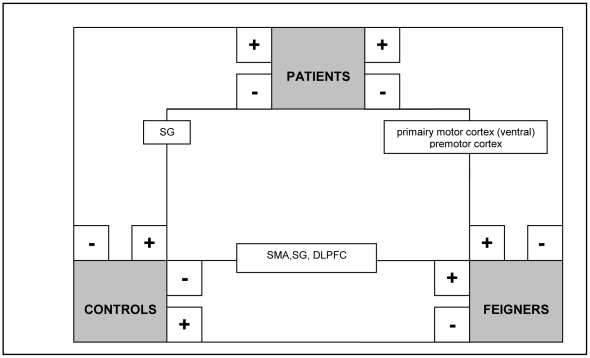
Schematic summary of significant clusters (P<0.05, whole-brain corrected at cluster level; k>200) in between-group comparisons (flipped data): movement imagery in the affected hand (analysis C). + = significantly more activated than -.

**Figure 5:**
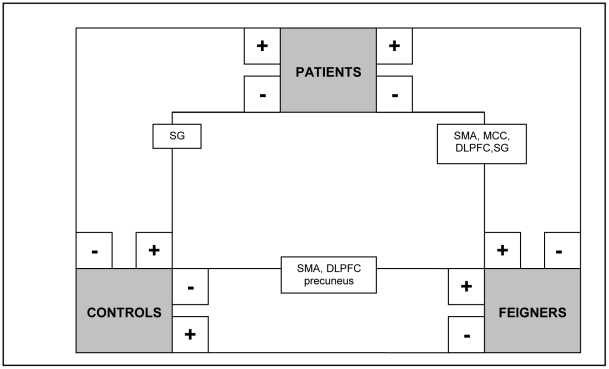
Schematic summary of significant clusters (P<0.05, whole-brain corrected at cluster level; k>200) in between-group comparisons (flipped data): movement imagery in the unaffected hand (analysis C). + = significantly more activated than -.

**Figure 6:**
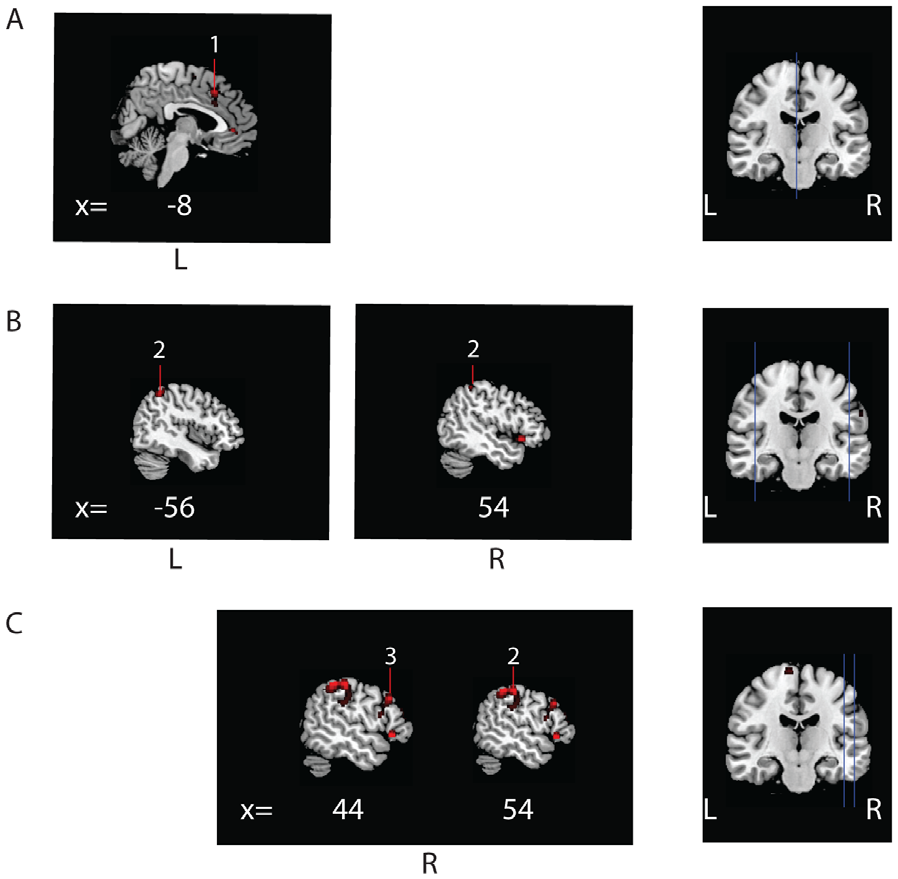
Significant hemisphere-specific cerebral activation patterns during movement execution in the affected hand for patients, feigners, and normal controls. Unflipped data for movement execution in the affected hand is shown for (A) patients versus normal controls, (B) feigners versus normal controls and (C) feigners versus patients. Figures were created with MRIcron, template Ch2better.nii.gz., without restriction of number of voxels. X-coordinates (in mm) for each saggital section are listed below. Numbers indicate clusters that were significant at cluster-level (P<0.05, whole-brain corrected at cluster level; k>200). Note that this Figure also displays activity (not numbered) significant at an uncorrected threshold, to preserve explorative qualities of the study. A: Increased activation in patients versus normal controls (red, t = 3.16 to yellow, t = 5). B: Increased activation in feigners versus normal controls (red, t = 3.16 to yellow, t = 5). C: Increased activation in feigners versus patients (red, t = 3.16 to yellow, t = 5). 1 = medial cingulate cortex, 2 = supramarginal gyrus, 3 = dorso lateral prefrontal cortex. L = left, R = right.

Conditions showed a complex pattern of significant activation (see Tables 5, 6, 7, and 8 and Figures 2, 3, 4, 5, and 6 for a complete overview of all significant results). Note that Figure 6 also contains activity significant at an uncorrected threshold of p<0.001 T>3.14 (see Figures 1 and 6).

**Table 5:** Cerebral activation related to movement execution of the affected hand (left-sided patients and feigners were flipped).

			contralateral (left)				ipsilateral (right)			
Contrast*	BA	k**	x	y	z	t/Z	x	y	z	t/Z
CP<NC										
1. frontal pole	10	209					28	64	2	5.6/5.3
2. ventral lateral prefrontal	45	364					50	38	14	4.4/4.3
3. precuneus	7	267	−10	−66	34	4.6/4.4				
4. cerebellum	IV–V	268					28	−42	−24	4.7/4.5
CP>NC										
1. ventral premotor cortex	6	788					52	4	26	5.8/5.4
2. supramarginal cortex	40	233					56	−46	28	5.7/5.4
3. superior temporal cortex	22	249	−48	14	−10	4.1/4.0	52	6	−8	4.9/4.7
4. anterior cingulate gyrus	32	575	−10	20	40	6.4/5.9	6	14	34	3.7/3.6
5. triangular cortex (inf frontal)	44	340	−52	16	8	4.2/4.0				
FC<NC										
1. prim. somatosensory cortex	1	781	−28	−36	72	5.4/5.0				
2. prim./sec. visual cortex	18/17	293	10	−58	2	5.5/5.2				
3. parahippocampal gyrus		222	−16	−13	−12	4.5/4.3				
4. cerebellum	IV–V	610					22	−32	−24	5.9/5.5
FC>NC										
1 a. suppl. motor cortex	6	9565****	−8	10	60	8.1/7.2				
Including premotor cortex	6	9565					40	10	46	
and superior temporal gyrus	22	9565	−48	14	10					
1 b. anterior cingulate gyrus	32	9565	−6	18	40	7.8/6.9				
2. DLPF	9	9565/515	−34	46	24	6.1/5.7	32	38	28	5.6/5.3
3. sup. frontal operculum***	44	9565/896	−54	12	30	4.2/4.0	54	8	0	5.9/5.5
4. supramarginal gyrus	40	1334/3191	−56	−26	28	6.9/6.3	60	−40	36	8.8/7.7
5. somatosensory ass. cortex	5	397	−22	−70	50	4.3/4.1	6	−70	40	5.3/5.0
6. nucleus caudatus	534	−8	4	10	4.8/4.6					
CP<FC										
1. supplementary motor cortex	6	677	−12	8	70	6.3/5.8	4	8	66	6.2/5.7
2a. frontal pole	10	893	−36	54	0	5.6/5.3	28	62	2	5.3/5.0
2b. vl prefrontal cortex	45	1944					42	38	16	5.3/5.1
2c. orbitofrontal cortex	47	1944					46	46	−2	5.8/5.4
3. supramarginal cortex	40	299–913	−52	−44	48	4.5/4.3	52	−48	46	5.6/5.2
4. precuneus	7	295	−10	−66	36	5.7/5.4	8	−66	36	4.7/4.5
5. superior parietal cortex	7	299	−32	−74	44	4.3/4.1				
6. frontal eyefields	8	316	−8	34	36	4.8/4.7	10	40	46	5.3/5.0

Coordinates refer to the voxels of maximum activation within significant clusters (P<0.05, whole-brain corrected at cluster level). Positive x, y, z coordinates (in mm) indicate locations right, anterior and superior to the middle of the anterior commissure.

*CP = conversion paresis, NC = normal controls, FC = feigning controls; BA = Brodmann area,

**k = contralateral/ipsilateral cluster extent, when separate clusters are involved;

*** = extending into insular cortex,

****large cluster contralateral

**Table 6:** Cerebral activation related to movement execution of the unaffected hand (left-sided patients and feigners were flipped).

			contralateral (left)				ipsilateral (right)			
Contrast*	BA	k**	x	y	z	t/Z	x	y	z	t/Z
CP<NC										
1. posterior cingulate gyrus	23	883	−20	−54	8	6.2/5.7				
2. inf parietal cortex/SG	40	357	−48	−42	26	5.1/4.8				
3. prim/sec visual cortex	17/18	505					26	−76	−4	5.7/5.4
4. cerebellar vermis		35					−16	−48	−26	4.2/4.0
CP>NC										
1. ventral premotor cortex	6	226					54	4	16	5.6/5.3
2. anterior cingulate gyrus	32	212	−10	20	40	5.1/4.9				
FC<NC										
1. cerebellum	VII	221	−8	−62	−48	4.6/4.4				
Extending into vermis		221					16	−60	42 4.2/4.0	
FC>NC										
1. dorsal premotor cortex	6	678					46	−8	50	5.3/5.0
2a. supplementary motor cortex	6	2449					0	8	62	5.7/5.3
b. cingulate gyrus	32/24	2449	−8	16	42	5.6/5.2	6	14	32	3.4/3.3
3. supramarginal gyrus	40	6224					52	−42	50	7.5/6.7
4. frontal operculum	44	420	−44	18	4	4.0/3.9				
5. inferior frontal cortex	47	775					44	2	−4	6.3/5.9
6. superior temporal gyrus	22	1505	−36	50	12	6.6/6.0				
CP<FC***										
1a. Dorsal premotor cortex	6	36370					34	−10	60 10.3/>8	
b. supplementary motor cortex	6	36370	−10	−8	64	10.1/>8	8	−4	72	10.2/i
c. supramarginal gyrus****	40	36370	−32	−44	40	9.8/>8	34	−40	52 10.7/>8	
d. frontal pole	4	36370					10	−22	76	7.4/6.7
e. prim. somatosensory cortex	3/2	36370					30	−30	48 10.2/>8	
f. superior temporal gyrus	22	36370					56	−46	14	7.6/6.8
g. retrosubicular gyrus	48	36370					58	−34	26 9.8/>8	
h. anterior cingulate gyrus	24	36370	−8	8	42	8.0/7.1	6	−2	44	7.7/6.9
fusiform gyrus	37	884	−46	−66	4	7.3/6.6				
posterior cingulate gyrus	23	273	−12	−26	38	6.3/5.9				
										
CP>FC										
1. frontal pole	10	256	−6	60	2	4.8/4.6				
2a.prim visual cortex	18	2005	−6	−64	10	5.0/4.8				
b.sec visual cortex	17	2005	−24	−46	−8	5.6/5.2				

Coordinates refer to the voxels of maximum activation within significant clusters (P<0.05, whole-brain corrected at cluster level, k>200). Positive x, y, z coordinates (in mm) indicate locations right, anterior and superior to the middle of the anterior commissure. BA = Brodmann area;

*CP = conversion paresis, NC = normal controls, FC = feigning controls;

**k = contralateral/ipsilateral cluster extent, when separate clusters are involved;

*** = large cluster contralateral;

****left supramarginal gyrus involved a separate cluster (kE = 5095).

**Table 7:** Cerebral activation related to movement imagery on the affected hand (left-sided patients and feigners were flipped).

			contralateral (left)				ipsilateral (right)			
Contrast*	BA	k**	x	y	z	t/Z	x	y	z	t/Z
										
CP<NC										
1. supramarginal gyrus	39/40	388	−50	−38	26	5.8/5.4				
CP>NC										
No significant results										
FC>NC										
1. supplementary motor cortex	6	597	−8	−14	58	4.9/4.6	12	−8	60	5.0/4.8
2. supramarginal gyrus	40	3817					52	−32	36	8.6/7.5
3. angular gyrus	39	214					44	−76	−12	4.4/4.3
4. a DLPFC***	9	240					30	44	36	6.2/5.8
b DLPFC***	9	418					36	16	46	4.7/5.5
5. pars triangular	45	311					52	2	4	5.3/5.0
6. primary somatosensory cortex	2	416	−58	−24	28	5.3/5.0				
7. somatosensory ass. cortex	5	328	−36	−44	64	5.8/5.4				
FC<NC										
No significant results										
CP<FC										
1. primary motor cortex	4	208					52	0	6	5.5/5.2
2. dorsal premotor cortex	6	390/310	−14	8	70	5.1/4.8	12	4	68	5.4/5.1
3. somatosensory ass cortex	5	1389	−38	−44	64	6.9/6.3				
4. superior temporal gyrus	22	1730					62	−42	18	7.6/6.9
5. associative visual cortex	19	306	−34	−76	32	5.2/4.9				
FC>NC										
No significant results										

Coordinates refer to the voxels of maximum activation within significant clusters (P<0.05, whole-brain corrected at cluster level). Positive x, y, z coordinates (in mm) indicate locations right, anterior and superior to the middle of the anterior commissure. BA = Brodmann area,

*CP = conversion paresis, NC = normal controls, FC = feigning controls;

**k = contralateral/ipsilateral cluster extent, when separate clusters are involved;

*** = two separate clusters.

**Table 8:** Cerebral activation related to movement imagery on the unaffected hand (left-sided patients and feigners were flipped).

			contralateral (left)				ipsilateral (right)			
Contrast*	BA	k**	x	y	z	t/Z	x	y	z	t/Z
CP<NC										
1. supramarginal gyrus	40	297	−46	−34	26	4.8/4.6				
CP>NC										
No significant results										
FC<NC										
No significant results										
FC>NC										
1. supplementary motor cortex***	6–8	5036					52	−2	4	4.7/4.5
2. a DLPFC***	9	274	−46	−40	58	5.5/5.2				
b DLPFC***	46	436					26	36	24	5.5/5.1
3.frontal eye fields	8	478					26	24	52	5.3/5.1
4. middle temporal gyrus	215	21	−54	−38	−6	5.5/5.2				
5. insula	43	242					32	−32	8	4.5/4.3
6. precuneus	7	242					52	−32	36	7.0/6.3
7. retrosplenial cingulate gyrus	29	263	−2	−42	12	5.3/5.0				
CP<FC										
1. cingulate gyrus and SMA****	6/32	14542	−10	−4	64	8/7.1				
2. DLPFC		45/46	340					56	8	0
3. DLPFC		46	751					36	40	28
4. parietal/occipital cortex	19–40	8489	−20	−82	−14	7/6.4				
including supramarginal gyrus	40	8489	−46	−34	−28					
5. insula	43	1025					40	−12	−12	5.1/4.9
CP>NC										
No significant results										

Coordinates refer to the voxels of maximum activation within significant clusters (P<0.05, whole-brain corrected at cluster level). Positive x, y, z coordinates (in mm) indicate locations right, anterior and superior to the middle of the anterior commissure. BA = Brodmann area,

*CP = conversion paresis, NC = normal controls, FC = feigning controls;

**k = contralateral/ipsilateral cluster extent, when separate clusters are involved;

*** = activation spreads bilateral and included frontal eye fields;

****spreads bilaterally.

#### Movement execution in the affected hand

In patients versus normal controls, increased activation was found ipsilateral to the affected side in the premotor cortex, supramarginal gyrus and cingulate cortex. Decreased activation was found in the ipsilateral prefrontal cortex and contralateral precuneus (Table 5 and Figures 2 and 6). In feigners versus normal controls, increased activation was found in the contralateral SMA and cingulate motor cortex, and bilateral increases were seen in prefrontal areas and supramarginal gyrus. In normal controls versus feigners, decreased activation was found in the prefrontal areas, and bilateral SMA, supramarginal gyrus and precuneus.

#### Movement execution in the unaffected side

Compared to normal controls, patients showed increased activity in the contralateral premotor cortex and ipsilateral medial cingulate cortex, while decreased activation was found in the ipsilateral posterior cingulate and supramarginal cortex (Table 6 and Figure 3). In patients versus feigners, patients showed increased activation in the ipsilateral frontal pole and a decrease in the contralateral premotor cortex, frontal pole, and bilateral SMA, supramarginal gyrus and cingulate motor cortex.

#### Movement imagery in the affected hand

Decreased contralateral supramarginal activity was found in patients compared to normal controls and decreased activations in the ventral segment of the primary motor cortex (including the ventral premotor cortex) and dorsal premotor cortex was found in patients compared to feigners (Table 7 and Figure 4). FC showed increased activity in SMA, supramarginal gyrus and prefrontal cortex compared to normal controls.

#### Movement imagery in the unaffected hand

The contralateral supramarginal gyrus was again decreased in patients versus normal controls, and now also versus feigners (see Table 8 and Figure 5). In patients versus feigners, decreased contralateral cingulate and ipsilateral SMA and prefrontal activity were found. Moreover, compared to normal controls, feigners again showed increased prefrontal and SMA, and now also precuneal activity.

### D. Conjunction analyses

Conjunction analysis (see Table 9) revealed underactivation of the ipsilateral ventral lateral prefrontal cortex during movement execution, and decreased activity in the supramarginal gyrus during movement imagery. This abnormal activity was specific for patients compared to both normal controls and feigners. On the other hand, patients and feigners both showed increases of activation compared to normal controls in the ipsilateral premotor cortex, supramarginal cortex and contralateral anterior cingulate cortex (motor area) and superior temporal gyrus.

**Table 9:** Cerebral activation in conjunction analysis (left-sided patients and feigners were flipped).

			contralateral (left)				ipsilateral (right)			
Contrast*	BA	k	x	y	z	t/Z	x	y	z	t/Z
Movement execution of the affected hand										
CP<NC & CP<FC										
1. ventral lateral prefrontal	45	265					48	38	14	4.4/4.2
CP>NC & FC>NC										
1. premotor cortex	6	204					40	−10	46	4.5/4.3
2. supramarginal cortex	40	233					56	−46	28	5.7/5.4
3. anterior cingulate cortex	32	548	−10	20	10	6.4/5.9				
4. superior temporal cortex	22	232	−48	14	−10	4.1/4.0				
Movement imagery on the unaffected hand										
CP<NC & CP<FC										
1. supramarginal gyrus	40	206	−46	−34	28	4.7/4.5				

Coordinates refer to the voxels of maximum activation within significant clusters (P<0.05, whole-brain corrected at cluster level). Positive x, y, z coordinates (in mm) indicate locations right, anterior and superior to the middle of the anterior commissure. BA = Brodmann area, k = cluster extent, CP = conversion paresis, NC = normal controls, FC = feigning controls.

*All other contrasts within the four conditions did not show significant results.

## Discussion

The direct comparison (i.e. not controlled for within-subject factors) of patients with feigners and normal controls reveals meaningful and innovative neurophysiological results. The combination of different tasks, control groups and statistical comparisons in the same dataset has resulted in a complex, but comprehensive, pattern of results. Most importantly, this is the first report of lateralized neurophysiological abnormalities, independent of whether patients had left- or right-sided paresis. A common neurophysiological view on conversion paresis can be extracted from this interrelated pattern of results. Furthermore, the specific hypotheses regarding specific neuroanatomical areas have been largely confirmed. See below for a detailed discussion of the results.

### Decreased prefrontal activation and willed action

The unintentional character of abnormal movement may be illustrated by decreased prefrontal activation (BA 46), which is lateralized in the right hemisphere (analysis B). It is independent of the side of paresis and has not been previously identified in conversion paresis by within-group comparison [10], [12]. This area has been reported to be involved in willed action based on free choice [5], [6]. Activation in this area also correlates with severity of trauma-related symptoms in Post Traumatic Stress Disorder (PTSD) [30]. Our finding of decreased prefrontal activation may seem at odds with the increased prefrontal activation reported by others [10], [12], [14], [15], but these effects should not be compared directly. This because the prefrontal increases reported elsewhere originated from within-group comparisons, which implied the methodological impossibility of detecting hemisphere-specific effects (see Introduction).

Also, when comparing the affected and unaffected side of patients (Analysis A), prefrontal areas 9, 45 and 10 are underactivated contralateral to the affected limb. This indicates symptom-related prefrontal underactivation. We propose that reduced prefrontal activity reflects the inability to perform consciously willed actions as they were intended by conversion paresis patients. However, this interpretation is at odds with the findings of others using the same statistical comparison. For example [10], described functional and effective connectivity between increased dorsolateral prefrontal activation and sensorimotor regions during motor imagery, which they interpreted as part of heightened self-monitoring and action-planning. On the other hand [13], described decreased prefrontal activation during movement execution similar to our within-group results during motor execution. It is important to consider the different types of task used in the studies; the activity in prefrontal areas may be increased in some behavioral tasks and decreased in others.

### Decreased parietal activation: a possible link between psychological factors and motor control

Flipped data indicate decreased parietal activation contralateral to the affected side, which was present in patients (analysis C) during both movement (supramarginal gyrus and precuneus) and imagery (supramarginal gyrus). In addition, unflipped data analysis reveal a right-sided decrease in supramarginal gyrus activation, independent of the side of symptoms (analysis B). It is a hemisphere-specific feature of conversion paresis, irrespective of symptom side, and we suggest that it reflects a major indicator of functional deficit in conversion paresis.

Underactivation of the supramarginal gyrus was previously reported by [12] in a patient with conversion paresis of the leg. The supramarginal gyrus is known to contribute to the integration of movement in relation to environmental and body information. With regard to purposeful hand movements, the supramarginal gyrus has been described as playing a pivotal role in prehension [31]–[33], which reflects its position at higher levels in the cerebral organization of movement. Prehension involves the incorporation of body scheme information and externally perceived shapes in the formation of adequate motor programs. Indeed, right supramarginal gyrus activity has been reported to increase after body scheme information was manipulated through stimulation of the median nerve in a single patient with conversion paresis. Moreover, this reaction was associated with a reduction of symptoms [34].

Lesions of the supramarginal gyrus are related to neurological conditions [35] such as visuo-spatial and ideomotor apraxia. These neurological disorders involve an unconscious and therefore unintentional inability to translate conscious motor plans into adequate movements, and a neglect of part of the environmental space and body scheme information.

We therefore propose that decreased supramarginal gyrus activity reflects impaired interaction of bodily scheme information and environmental cues, resulting in ineffective movement initiation.

The contralateral precuneus is underactivated in the affected hand in patients, specifically during movement execution (analysis C) and also present when comparing intentional with unintentional paresis. This indicates that it is not the result of abnormal movement in general, but may be specific for the unintentional nature of conversion paresis. In this respect, increased precuneus activation in feigners (during imagery of unaffected hand movement) may reflect the opposite effect, i.e. increased attention to *intended* movement. Decreased precuneus activation in conversion paresis may provide an important link with psychological mechanisms accompanying intentionality.

In a review on the precuneus [36], four behavioral correlates of precuneus activity based on fMRI and PET findings were described: episodic memory, self-processing, consciousness, and spatial attention or visuo-spatial imagery. The latter, spatial attention and visuo-spatial imagery, would be consistent with the abnormal preparatory processes described above. In our data, however, reduced precuneus activation is not present during imagery. Other precuneus functions therefore deserve attention. Its posterior part (in conjunction with the cingulate cortex and prefrontal regions) has been implicated specifically in the retrieval of autobiographic memories (episodic memory), but not in semantic memory in general. Furthermore, the anterior precuneus and the interconnected medial prefrontal regions are implicated in self-processing tasks. For example, the precuneus is activated when subjects are required to judge between self-relevant and self-irrelevant traits. Finally, the precuneus is involved in consciousness: it shows increased activation during a default conscious resting state (that decreases during goal-directed behavior) and during alternative states of consciousness such as slow-wave sleep, rapid-eye movement sleep, hypnotic state [37], [38], pharmacologically induced general anesthesia and persistent vegetative state.

Failing autobiographic memory, personal disintegration and altered states of consciousness (hypnoses) were described in the first theories of conversion disorder [2], [39]. With these conditions, patients are reluctant in the retrieval of episodic memories, show lack of self-insight or personal integration (i.e. dissociative symptoms) and with respect to their level of consciousness they show states comparable to hypnosis. In current neurocognitive models of conversion disorder, some scientists still use the concepts of hypnosis [40], autobiographic memory and dissociation [3].

Contrary to our results, a n = 1 study comparing conversion paresis to hypnotically induced paresis and normal movement by normal controls showed increased precuneal activation contralateral to the affected side [17]. This was absent when using the unaffected limb. This finding was based on a within-group comparison during execution, and was not replicated by our data, in which no abnormal precuneal activation was found in the within-group contrast. A possible explanation for this inconsistency may be the type of task. While another study [22] used a go-no go task including a preparation cue, we did not use any cues. The authors made a distinction between (1) hypnotic states that were induced without specific suggestion, which was related with reduced precuneus activity, and (2) hypnotic states induced by specific suggestion and related with increased activity. They elegantly stated that the precuneus may be involved in the envisioning of future events from a first-person perspective. This theory can be applied both to a failure of conscious internal control over motor function (decreased precuneus function in conversion paresis) and to intensified unconscious – but internal – control over motor function (increased precuneus activity during hypnosis). The authors suggested that the precuneus may be part of a network at work in self-monitoring processes. In this respect, the precuneus may be involved in the disturbed unintentional internal representations and memories related to the self.

Our results and the current theories on the role of the precuneus in conversion paresis seem to imply that the precuneus is not specifically involved in conversion *paresis*, but that it may be involved in other types of conversions disorder as well. However, most motor conversion symptoms are difficult to study with neuroimaging techniques, since excessive movement interferes with data acquisition in fMRI. Nevertheless, conversion tremor has been studied using fMRI in 8 patients [41]. Interestingly, in that study the precuneus was part of an abnormal functional network and was interpreted as part of the psychological experience underlying the involuntary character of conversion movements. In conversion conditions with both involuntary additional movement (tremor) and lack of movement (paresis), impaired precuneus activation thus appears to play a role.

In patients with PTSD, decreased precuneus activity was recently identified during the resting state and was interpreted as reflecting altered self-perception and consciousness [30]. Moreover, the severity of trauma-related symptoms correlated with posterior cingulate/precuneus and right DLPFC (BA 46) connectivity (see above for BA 46 in conversion paresis). We propose that decreased precuneus functioning in conversion disorder is elicited by overwhelming personal experiences, such as psychological trauma. However, the reverse could also be true. Pre-existing underactivation of the precuneus may cause patients to react differently to psychological trauma. But if this were the case, one would expect to find decreased precuneus activity independent of symptoms and symptom lateralization. Our data should have shown this in unflipped data and imagery conditions, but this is not the case. This may imply that precuneus underactivation is associated with the process of psychological trauma manifesting into overt physical symptoms.

### Cingulate cortex, premotor cortex and SMA: different movement preparation in patients and feigners

Abnormal parietal and prefrontal function in the higher stages of motor control may result in abnormal function in the later stages of motor control, i.e. motor areas and cingulate cortex.

Conjunction analysis (D) suggests that patients and feigners both show abnormal movement preparation, but explorative comparisons (analysis B) – not controlled for within-subject factors – suggest they do so in different ways: we found different movement preparation correlates in patients compared to feigners during abnormal movement. Patients showed increased premotor activation, while feigners showed increased pre-SMA activation.

The cerebral activation patterns of patients and feigners suggest a difference between the groups with respect to (a) the degree of free selection of movement in space compared to normal movement and (b) the degree of internal versus externally guided abnormal movement. Note that both patients and feigners received paced instructions on movement from the experimenters and were therefore *externally guided* by the experimenter. However, both groups moved abnormally and therefore did not follow the instructions on the screen, while the normal controls did. This resulted in a larger ‘freedom of space’ in movement output in both patients and feigners. In conversion paresis, this is believed to be caused by an unidentified unintentional process, while feigners intentionally produce abnormal movement. These differences in *intentionality* and subsequent internal initiation of movement may be explained by differences between feigners and conversion paresis in the *preparation* phase of movement. Patients showed increased premotor activity. This may indicate a greater preparatory effort required to initiate movement compared to normal controls. Interestingly, the increase in premotor activity was *ipsilateral* during movement execution in the affected hand, and *contralateral* during movement execution in the unaffected hand. Since data were flipped, this does not indicate the presence of a hemisphere-specific effect, but it does indicate increased premotor activation contralateral to the affected hand, regardless of which hand was moving. In contrast, the SMA is specifically overactive in feigners compared to normal controls (see also [14]). Although results are not consistent [42] and should not be oversimplified [43], it has been proposed that the (pre-)SMA is relatively more involved in learning *internally* guided movements, while the premotor cortex is thought to be more involved in *externally* guided movements [44], [45].We therefore propose that increased pre-SMA activation in feigners suggests that they were more internally driven to alter their movements of the ‘affected’ hand compared to patients.

Contrary to expectations, the previously reported anterior cingulate overactivation was not found in our within-group comparisons of the affected and unaffected side before the threshold was substantially lowered. The different nature of our assigned task may be relevant here, since previous work studied imagery of hand rotation (see also [10]), sensory processing [15] or motor preparation [12] rather than actual movement, as in the present study. In more general terms, the early theories on active inhibition of motor commands by cingulate overactivation have not been supported by consistent cumulative evidence throughout different paradigms and subjects. Recently, a high-impact study on feigned paresis and hypnotically induced paresis also reported no evidence for active inhibition of motor commands mediated by cingulate overactivation [17]. Another study of cingulate overactivation revealed inconsistencies between neuroanatomical function and the interpretation of initial neuroimaging results in conversion paresis [20].

In our data (analysis C), we found increased activity of the *medial* part of the cingulate cortex – referred to by [46] as the cingulate motor zone – in both patients and feigners.

According to a subdivision of cingulate areas proposed by [7], [8], this area is referred to as the aMCC. The aMCC is involved in complex arm movements, and is consistently activated in relation to the internal selection of movement [46]. Previous results comparing fixed movements with freely selected movements indicated that increased aMCC activation is present [5] during the *free selection of a target in external space* (environmental target) in comparison to a *cued* target in space. Our patients and feigners had greater freedom in the selection of movement compared to normal controls since they were unable to follow exact instructions, or feigned this inability, while the controls followed the instructions. In our data, this result is not specific for conversion paresis but is also present in feigners. We therefore propose that cingulate overactivation reflects a similar phenomenon in both intentional and unintentional paresis, which is related to abnormal movement output (made in external space).

### Limitations

The findings discussed above are based on a rather unconventional methodological approach. The explorative character of this study was used to reveal cerebral activation patterns that remained hidden in previous studies for reasons described in the Introduction. However, this explorative approach entails its own methodological issues, such as the problems of multiple comparisons in relatively small sample sizes. In this section, we discuss these limitations.

#### Multiple comparisons

When performing four types of statistical comparisons with three groups and four task conditions, the problem of multiple comparisons inevitably appears. We attempted to minimize the chance of Type I errors by only reporting results significant at cluster level (see below) and by limiting the discussion and interpretation of our results to those significant clusters that were relevant to the initial hypotheses. Although we are keenly aware of the increased chance of Type I errors in this study, we believe that our results are a crucial step in understanding conversion disorder. The aim of this study was to explore the commonalities in previous studies – despite the diverse methodology – in order to formulate a more general hypothesis about the neurophysiological mechanisms of this disorder. The methodology used in our study minimizes the risk of Type II errors, such as we detected in previous research. This is also important in such a complex and poorly understood neuropsychiatric condition. However, we certainly emphasize the need for a second round of research with experimental designs that formally test the findings in our study.

#### Small sample sizes

Due to the narrow inclusion criteria needed to perform adequate neuroimaging contrasts, we were only able to include 9 patients. The small sample size of the patient group decreases power. This group contained both right- and left-sided paresis, which further complicated the sample. One could argue that non-parametric analyses are needed (in unflipped data) to exclude the possibility that outliers have influenced the results. However, by applying a model that included both subgroups in each comparison, we have attempted to increase the power by limiting the influence of individuals. An additional problem with small sample sizes is the high error variance. We addressed this problem by pooling the error variance of all 43 subjects together to increase the power. Furthermore, inspection of individual results did not reveal any relevant outliers. To deal with the small sample sizes, we used a 2×5 ANOVA in analyses A and B with unflipped data. A problem with flipped data is that it is assumed that right- and left-sided paresis will show identical effects – i.e. equal variances (see the Introduction for other reasons to perform analyses with unflipped data). We allowed for a possibly different effect size for left- and right-affected groups (at the cost of two degrees of freedom) by initially defining five groups instead of three. (Note that this minimizes error variance.) Therefore, two-way between-group ANOVA was used. Factor 1 (group) contained five levels (normal controls, right feigners, left feigners, right patients and left patients), and factor 2 (condition) contained four levels (Execution affected, Execution unaffected, Imagery affected, Imagery unaffected). Note that this ANOVA design does *not* implicate that five groups were used separately, with even smaller sample sizes of only 4, 5, 6 or 7 subjects per group. When contrasts were defined, left- and right-sided groups were merged to limit the number of comparisons. Thus, in all four types of statistical analyses (A, B, C, D), *three* groups were included, with group sizes n = 22 for normal controls, n = 13 for feigners and n = 9 for patients.

#### Complex set of results

The inclusion of three groups, four tasks and four types of statistical analyses resulted in a large amount of data, which has been complex to analyze. We therefore decided to describe a selection of anatomical areas (in the Results and Discussion) based on the hypotheses described in the Introduction. However, a complete overview of significant activity (P<0.05, whole-brain corrected at cluster level; k>200) is shown in the Tables for readers who are interested in more explorative data. Moreover, Figures 1 and 6 display activity that is significant at an uncorrected threshold (p<0.001).

#### Different task instructions

Patients and feigners received different instructions and performed different tasks. For example; patients tried to move, but could not do so adequately, while feigners tried not to move adequately. Also, compared to patients, the feigners were performing a dual task. One could argue that these differences influenced the fMRI results. Indeed, these differences between the groups limit the interpretation of our results. But the explorative character of this study has also generated highly interesting hypotheses for future research. For example, an important difference between feigning and conversion paresis is the lack of self-agency [47] and mismatch detection. While the feigners are indeed moving abnormally, they are doing so with an intact motor plan, consistent proprioceptive feedback and therefore have matching information at the comparator region, which leads to an intact sense of agency for their feigned movement. In contrast, the conversion paresis patient has an intact motor plan, but proprioceptive feedback is inconsistent with the action they are attempting to perform. Therefore a mismatch at the comparator region (right temporoparietal junction) [48] may occur. Although we reported underactivation in the right parietal lobe (as did Voon et al. [41]), and not the increased activation that might be expected in the case of mismatch, the question of agency should be addressed in future research.

Analyses B, C, and D compared the affected sides of different groups, but without controlling for experimental confounds such as differences in anxiety or depression, medication or illness duration. This design was used to isolate the cerebral correlates of these aspects, even though some experimental psychologists regard them as confounds. After all, one of these ‘confounds’ probably contains the unique feature of conversion disorders, which is not present in feigners or hypnotically induced paresis. By understanding this aspect, we increase our understanding of the etiology of the disorder. The current results show cerebral areas that are related to the general differences between groups, which may indeed be factors such as depression. In combination with current knowledge of the functional neuroanatomy, and within a theoretical framework [3], these results have led to a description of an initial, tentative neurophysiological model of conversion disorder.

### Conclusions: a neurophysiological view of conversion disorder

In the case of conversion paresis, we propose that decreased supramarginal activation reflects the development of paresis rather than other conversion symptoms, resulting in subsequent abnormal, internally generated, movement initiation processes. These processes may, in turn, be reflected by abnormal motor preparation in the premotor cortex and cingulate cortex. Decreased prefrontal activity may reflect the unintentional nature of conversion paresis in combination with the precuneus.

Symptoms appear to be produced unintentionally due to impaired willed action. Further research that includes attention for precuneal functioning is warranted and should reveal an interesting relationship between psychological and physical symptoms.

We conclude by presenting a hypothetical and integrative neurophysiological view on the neurophysiological substrates of conversion disorder based on current findings about conversion paresis. After all, different types of conversion disorder may share a similar neurophysiological background, and it remains unclear why patients develop different types of symptoms. First, we propose that decreased precuneus activity may be a result of the unintentional influence of psychological stressors and should therefore be causally related to the development of all forms of conversion symptoms. Second, specific forms of conversion disorder might show matching cerebral correlates. Our observations of decreased activity in the supramarginal gyrus appear to fit clinical and theoretical knowledge on *motor* preparation, in combination with other structures within the hierarchical organization of motor control. We therefore propose that the supramarginal gyrus is responsible for the particular *type* of conversion reaction studied here: conversion paresis. Studies on other forms of conversion symptoms appear to support this idea. Other types of conversion symptoms probably include additional brain areas that are functionally associated with the specific symptom. Indeed, somatosensory cortical activity is decreased in conversion sensory loss [49], [50] and the visual cortices show reduced activity in psychogenic visual loss [51]. Also, in one out of four studies available on other types of conversion disorder, symptom-associated decrease of posterior parietal areas has been reported [49] (note that two out of four reports used ERP and ROI analysis that did not include the precuneus, while the fourth paper is unclear about the contrasts that were used).
